# Prognostic Value of Microvascular Density in Dukes A and B (T1–T4, N0, M0) Colorectal Carcinomas

**DOI:** 10.1155/2009/679830

**Published:** 2009-11-04

**Authors:** Rafael Uribarrena A, Javier Ortego, Javier Fuentes, Nuria Raventós, Pilar Parra, Rafael Uribarrena E

**Affiliations:** ^1^Digestive Service, Hospital Universitario Miguel Servet, 50009, Zaragoza, Spain; ^2^Department of Pathology, Hospital Clínico Universitario Lozano Blesa, 50009, Zaragoza, Spain; ^3^Department of Emergency, Hospital Universitario Miguel Servet, 50009, Zaragoza, Spain

## Abstract

*Background*. Aproximatelly 30% of patients operated on for colorectal cancer (CRC), with an expectedly favourable prognosis (Dukes A-B/T1–T4, N0, M0) suffer recurrence and/or die. *Method*. In order to determine if tumor microvascular density (MVD) is a prognostic factor in CRC, samples from tumors of 104 Dukes A-B CRC patients were retrospectively studied. Immunohistochemistry was performed for anti-CD34 antibody to visualize tumor vascularisation. MVD was expressed as the total number of vessels and as the percentage of microvascular area. We calculated MVD with a morphometry program and performed descriptive, bivariate, and survival statistics. *Results*. The mean number of vessels was 37.37/200x field, and the mean vascular area was the 3.972%. 30% of the patients with < 37 vessels/field, and 21% of the patients with > 37 vessels/field, experienced recurrence/death. The 35% of patients with < 4% of vascular area died following recurrence, compared with 14% of patients with ≥4% of vascular area. These differences in % of vascular area were statistically significant. *Conclusion*. MVD expressed as the total number of vessels had no a statistically significant influence on the evolution of CRC. However, neoplasias with a greater % of vascular were associated to a better outcome.

## 1. Introduction

Despite the advances in diagnostic and therapeutic methods, the global survival rate for colorectal adenocarcinoma (CRC) is approximately 50% and has not substantially improved in recent years [1]. It ranges 80%–90% in Dukes stage A, 60%–80% in Dukes B, 30%–35% in Dukes C, and 5%–25% in Dukes D [2–5]. The main prognostic factor in CRC is the presence or not of metastasis in the regional lymph nodes [1, 6]. Paradoxically, 20%–35% of patients diagnosed with CRC with negative lymph nodes (N0) based on conventional histological techniques will suffer tumour recurrence within 5 years [4, 5]. This leads us to ask what other possible prognostic factors might influence the evolution of the disease.

The degree to which a tumour is vascularised, expressed as microvascular density (MVD), also influences the evolution of CRC. In the majority of solid tumours, angiogenesis is essential for tumour growth and for its locorregional and remote extension [7, 8]. Initially, tumour growth is independent of the vascular contribution, as the neoplasic cells receive nutrition from the diffusion of oxygen and from the nutrients in the surrounding tissues. This characterises the prevascular phase of neoplasic growth, during which the tumour is only a few millimetres in size and has little capacity to disseminate. Subsequently, angiogenic substances are released by the tumour cells and healthy host tissue (fibroblasts, endothelial cells, macrophages, plasma cells). The angiogenic stimuli are varied and interrelated. They include hypoxia, specific growth factors, and interleukins [7, 9–11]. Following the release of the angiogenic substances, the endothelial cells migrate towards the tumour and proliferate inside of it, forming capillaries. The newly formed vessels are more porous, with fragmented basal membranes that allow nutrients to pass to the tumour stroma and cancer cells to enter into the circulation. In this vascular phase, the neoplasia grows quickly and the risk of remote metastasis increases [7, 12]. Nevertheless, tumor vascularisation is but a single step in the metastatic process. For remote metastasis to occur, each of the independent steps of the process must take place [13]. Furthermore, the increase of the tumour vascularisation permits the entry of immune system cells, that could limit tumor development.

There are various studies on the relationship between vascular density and other tumor parameters such as survival and remote metastasis in tumours of the breast and prostate gland, melanoma, and large-cell pulmonary carcinoma. In most cases, a direct relationship was found between MVD and metastasis, and an inverse relationship was found between MVD and patient survival [14, 15]. More recently, similar studies have been performed on colorectal cancer (CRC). The results of those studies are controversial because although the majority of them reported an association between a greater microvascular density, remote metastasis, and patient survival [16–28], others did not [29–31], and in some cases, high MVD was associated with better prognosis [32–35].

Perhaps because of this contradictory results and although the majority tend to consider high MVD as a negative prognostic factor, a consensus review performed by the College of American Pathologists in 1999 on the prognostic factors in CRC classified MVD as a prognostic factors that required further study (category III) [36].

## 2. Methods

### 2.1. Patients

The retrospective study included 104 consecutive patients diagnosed with CRC in Dukes stages A and B (T1–T4, N0, M0) in the University Clinic Hospital in Zaragoza, Spain, and in the Provincial Hospital of Zaragoza. Exclusion criteria were as follows: (1) the administration of adjuvant radio and chemotherapy, (2) lymph node metastasis detected by means of the conventional histological methods (i.e., H&E), (3) remote metastasis at the moment of surgery, and (4) other neoplasias detected 5 years before or after CRC diagnosis that could interfere with its evolution.

### 2.2. Immunohistochemistry

**Figure 1:**
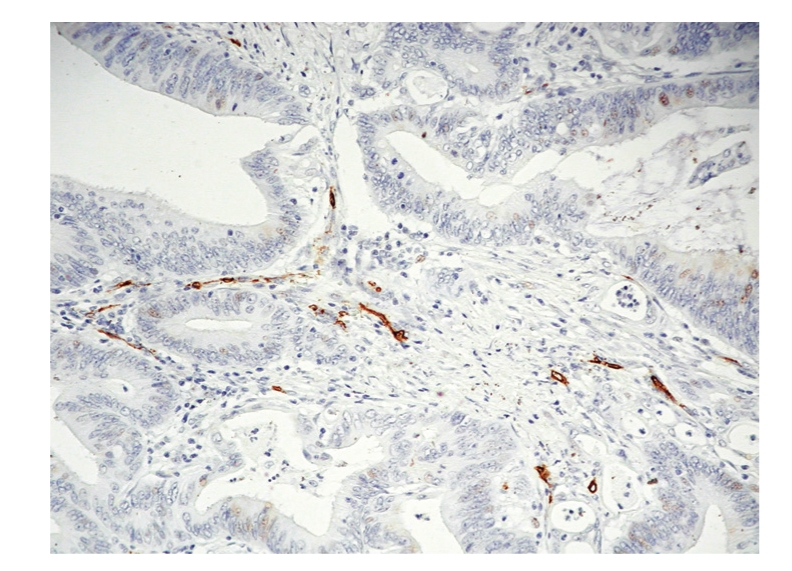
Low microvascular density foci.

**Figure 2:**
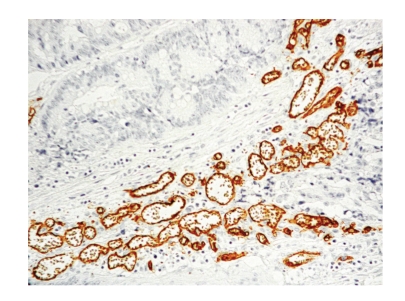
High microvascular density foci.

Tissue samples from each CRC case were fixed in 10% neutral formalin and embeded in paraffin. The most representative portion of a tumour was selected for processing. Five micron thick sections of tissue were deparaffinised, hydrated, and placed in citrate buffer (pH 6). Endogenous peroxidise activity was blocked of incubation in 5% H2O2 in methanol. Antigen was demasked by pressure boiling in citrate (Target Retrieval Solution, Dakocytomation, Glostrup, Denmark) for 10 minutes. In order to ascertain tumour MVD, samples were blocked with horse serum and then incubated in the cross-sections through blocking serum (horse serum) and antiHuman Antibody CD34 (1/0 Ac monoclonal, clon QBEND/10 Dakocytomation, Glostrup, Denmark), since blood vessels are CD34 positive (Figures 1 and 2, foci of low and high MVD). We detected the antibody with the EnVision kit (Dakocytomation), with diaminobenzidine as a developer, and nuclear contrast staining with haematoxylin, as a chromogen.

### 2.3. Microscopic Study

The histological sections, immunostained for AB CD34, were studied by two pathologist (JO, PP), with a Leica DM 4000B microscope at a semipanoramic focus of 10× in order to locate the tumoral areas that contains the greater concentration of blood capillaries (hot spots).

**Figure 3:**
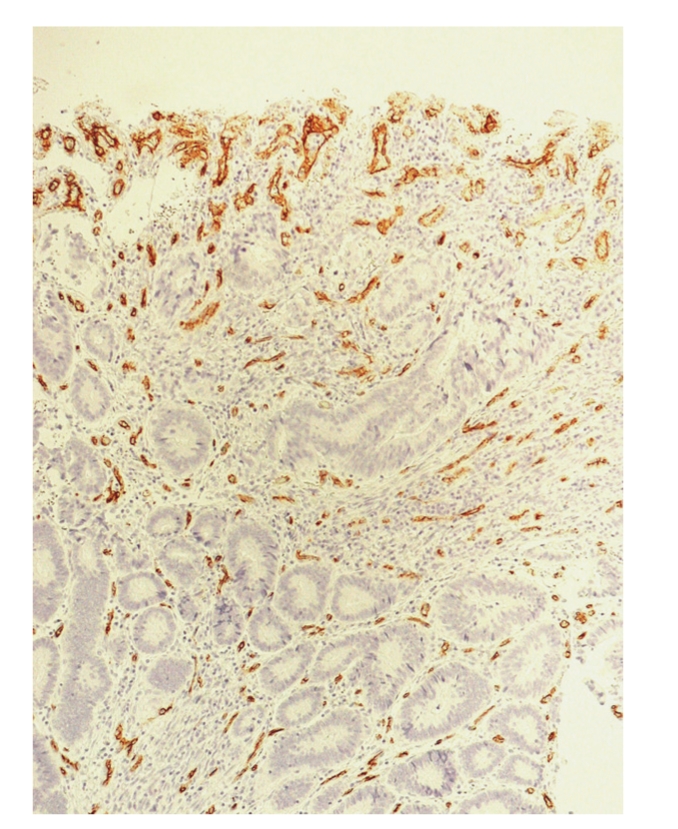
High MVD in superficial ulcerated area.

Hot spots were selected by discarding all of the zones in which an increase in MVD was not intrinsically dependent on tumour growth dynamics but on added circumstances and/or complications and could be considered as reactive (Figure 3). The ruled out zones were superficial erosion/ulceration, the circumferential margin of intratumour necrosis foci, and host-tumour interface.

Images of eight selected fields within the hot spots were captured at 200× (ocular 10×, objective 20×) with a Leica DFC 480 digital camera. The images were archived serially, ignoring the characteristics of individual CRC cases (e.g., evolution).

**Figure 4:**
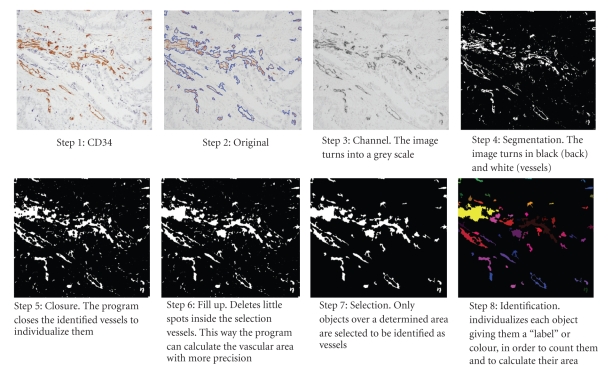
Automatic vessel counting with *ContImUZ 1.0* morphometry program the whole process.

MVD was measured by counting the number of blood capillaries/200× field. Well-formed vascular structures, rudimentary vascular structures, and angioblasts, (i.e., all those structures that were inmunoreactive for CD 34) were included. The area of the vascularisation (cumulative area of each and every one of capillary vessels compared with the total area of the all field) was quantified relative to the total tumour area of each imaged field (percentage of area). MVD quantification was performed with *ContImUZ 1.0* morphometry program, designed by the Digital Image Treatment Centre at the University of Zaragoza, that automatically calculates the Microvascularisation Density (MVD) of the tumour, by counting the number of objects in the image, by means of an automatic function with manual connection (Figure 4, whole process).

### 2.4. Statistical Study

Descriptive statistics of tumor characteristics included local infiltration: submucosa (T1), muscular (T2), meso (T3), and serosa-adjacents organs (T4); Dukes stage A or B (C and D were excluded); vascular, lymph node, and perineural invasion (yes or no); and MVD: expressed as the total number of vessels and as percentage of the vascular area; we also included tumour recurrence (yes or no) and month of recurrence and death (yes or no) and month of death.

Bivariate inferential statistics were performed to identify differences between the patients with a high and low MVD. We divided the patients in 2 groups according to the number of vessels per “hot spot” and percentage of vascularised area. As a cut point we used the arithmetic mean (> or <37 vessels > or <4% of vascular area), relative to the studied variables. Pearson's chi-squared test was used, with the Yates correction or Fisher's exact test when necessary. The Student *t*-test, variance analysis, the nonparametric Mann-Whitney U test, and the Kruskal-Wallis test were also used, for cases in which the ANOVA or the Kruskal-Wallis results were significant, between-group analyses using the Pearson correlation coefficient and the Spearman rank coefficient to ascertain which variables accounted for the differences. A 95% confidence level was chosen for statistical analyses.

## 3. Results

Of the 104 cases studied, 56 were Dukes stage A and 48 were Dukes stage B. According to local infiltration, 11 patients were T1, 45 were T2, 32 were T3, and 16 were T4.

The mean number of vessels per hot spot was 37.37 (range 14.9–74, standard deviation 9.75, median confidence interval 35.11–39.63), and the mean vascular area, expressed as % of tumor area occupied by vessels, was 3.976% (range 1.155–16.111, standard deviation 1.909, median confidence interval 3.534–4.419).

In 28 cases (26.92%) there was recurrence of the disease led to death. Recurrence occurred at a mean of 23.73 months of follow-up, (range 2–82 months, standard deviation (SD) ±20.24 months, mean confidence interval (CI) 14.75–32.7). The bivariate inferential statistics was used to compare the total number of vessels and the vascular area relative to the Dukes stage, the local invasion (T stage) and the occurrence of recurrence and death.

The results of the Kruskal-Wallis demonstrated that there were not significant differences in the number of vessels (*P* = .918, > .05) and in the percentage of the tumor vascularised area (*P* = .635, > .05) compared with the local invasion (Tables 1 and 2).

We evaluated vascular invasion by neoplastic cells as a prognostic factor in CRC. Neoplastic cell invasion was detected in 5 of the 104 patients studied, and recurrence occurred in 4 (80%) of those patients. Recurrence occurred in 24 (24.24%) of the remaining 99 patients. According to Fisher's exact test to compare vascular invasion (No, Yes), with cancer recurrence and death (No, Yes), we found significant differences between two groups. Patients with tumour vascular invasion had a significantly higher risk of recurrence and death (*P* = .018).

We also compared the Dukes stage with the tumor recurrence and death. In the Dukes A group (*n* = 56), 8 patients (14.3%) died due to recurrence of the disease. In Dukes B group (*n* = 48) cases, 20 patients (41.7%) died due to the CRC. Chi-square test indicated that there were significant differences between the Dukes A and Dukes B patients, with Dukes B patients having a higher risk of CRC recurrence (*P* value = .004). If we analyze local invasion, survival rate in T1 was 90.90%, in T2 84.44%, in T3 62.50%, and in T4 50.0%. T3 and T4 colorectal cancers had a significant association with tumor recurrence and death (*P* value = .0446) (Tables 3 and 4).

The bivariate inferential statistics of the number of vascular objects (<37, ≥37) and tumor recurrence and death showed that there were no group differences regarding recurrence and death. Survival rate was 70.1% in <37 vessels per area group and 78.4% in >37 group. Pearson's Chi square test indicated that there were no significant group differences in survival relative to the number of vascular objects (*P *value = .5000) (Table 5).

However, the bivariate inferential statistics for the vascularised tumour area (<4%, ≥4%) and the colorectal cancer recurrence and death showed that recurrence in <4% group was 34.9%, compared with the 14.6% in the ≥4% group. Pearson's Chi-square test demonstrated that there was a significant group difference in the survival relative to the vascularised tumor area. Patients with higher vascularized tumor area had a significant association with a better outcome (*P *value = .040) (Table 6).

## 4. Discussion

Other authors have previously reported that the main prognostic factor in CRC is the presence or absence of tumour metastasis in the regional lymph nodes [12, 13]. A decrease in CRC rate was observed between patients with Dukes stages B (N0) and Dukes stage C (N1), with survival rates of approximately 60%–80% and 30%–35%, respectively [2, 3]. It is especially notable that about 30% of the patients that are lymph node negative, based on routine H&E analysis, die from recurrence of the disease [2–5]. In this study, 14% of the patients with Dukes A CRC and 41% with Dukes B CRC suffered tumour recidivism that led to death. This represents almost a 27% recurrence for the Dukes A and B groups.

Dukes stage and local tumour growth are contrasted and widely accepted prognostic factors in CRC [2–5, 37, 38]. As expected, a significantly higher frequency of recidivism and death was observed in cases classified as Dukes B (41%) than Dukes A (14%).

We also examined the association between vascular invasion and prognosis. The finding of neoplasic cells in the blood vessels of the tumour was associated with an increase of tumour recurrence and survival reduction. Blood flow to the tumour is necessary for remote metastasis to occur and our finding agrees with the majority of literature pertaining to CRC prognostic factors [39–42].

There are several studies with contradictory results regarding MVD as a prognostic factor in CRC. In the majority of reports, increase in tumour vascularisation favours the growth and remote dissemination of the cancer cells and is, therefore, a predictor of poor outcome in CRC [16–28]. On the other hand, some authors have reported higher survival rates in tumours with high MVD. According to those studies, a higher MVD would induce a greater immune response that could slow tumour growth and dissemination [32–35]. Finally, other published studies reported no relationship between tumour vascularisation and the evolution of CRC [29–31].

One of the advantages of immunohistochemistry is the possibility of using tissue samples preserved in paraffin, which permits retrospective studies, as in our case [43]. To evaluate tumour vascularisation, MVD was determined by calculating the total number of vessels (number of objects) in the areas of greatest CD 34 immunoreactivity, that is, the areas with the higher number of capillary vessels or “hot spots.” Using this technique, the total number of vascular elements was considered, without differentiating their size. We also evaluated the MVD by determinating the percentage of the total tumor area that was vascularised. In that way, it was possible to differentiate microvessels from macrovessels, which gave us a more precise measure of the true degree of vascularisation. As a cutoff point for the survival study, the arithmetic mean of number of vessels (37/field) and vascularised area per “hot spot” (4%) of all patients was used. In order to increase accuracy, the number of vessels and the percentage of vascular area were evaluated in eight hot spots.

Compared with other studies, the present study introduces three novel approaches. Firstly, we calculated the number of vessels and the vascular area with a semiautomatic procedure (digital photography with a* Leica DFC 480 *camera and image analysis with an automatic quantification program* ContImUZ*). In this way, accuracy and objectivity were increased compared with semiquantative calculation which quantifies MVD as low, medium, or high [21, 23, 24]. Secondly, the area of vascularisation may better represent the true extent of tumor vascularisation, as considering only that the total number of vessels may induce a skewed result. That is, many of the vessels may be of scant calibre, and the MVD may be, in fact, much lower than calculated. Finally, “hot spots” were always chosen from fields away from the tumour margins, the host-tumour interface, areas of superficial erosion/ulceration, and the circumferential margin of necrosis foci. Those areas usually show increased vascularisation relative to the reminder of the tumour and may provide an erroneous idea of the true MVD.

Analysing the results of the bivariate analyses in relation to the total number of vascular objects, we observed that almost 30% of the patients with <37 vessels/field experienced tumour recurrence that led to death. This was the case for 21% of the patients with >37 vessels/field. However, the difference was not statistically significant. Therefore, we concluded that the number of vascular objects in the fields of greatest vascular density is not a prognostic factor in CRC. We observed a significant difference in recurrence and survival relative to the MVD expressed as % of vascular tumor area (% of tumor area occupied by vessels). Thirty-five percent of patients with <4% vascular area died following tumour recurrence compared with 14% of patients with ≥4% vascular area. The patients who survived with no recurrence had a significantly larger vascularised area compared with patients who had a poor evolution.

In conclusion, no significant relationship was observed between MVD, expressed as number of objects and tumor recurrence and death. However, there was a significant statistical association between a higher % of vascular tumor area and a more favourable prognosis. Dukes stage, local infiltration, and vascular invasion by neoplastic cells can also be considered as prognostic factors in CRC.

## Figures and Tables

**Table 1:** Number of vessels relative to local invasion.

	Number of vessels		*P*-value
	*N*	*Mean*	*Kruskal-Wallis*
Submcosa-T1	11	37.29	
Muscular-T2	45	36.40	.918
Meso-T3	32	38.43	
Serosa-T4	16	37.98	

**Table 2:** Percentage of tumor-vascularised area relative to local invasion.

	% vascularised area		*P*-value
	*N*	*Mean*	*Kruskal-Wallis*
Submcosa-T1	11	3.966	
Muscular-T2	45	3.687	.635
Meso-T3	32	4.421	
Serosa-T4	16	3.981	

**Table 3:** Dukes stage relative to recurrence-death.

DUKES	Recurrence-death			*P*-value
	No	Yes	*Total*	*Chi-square*
A	48	8	56	
	85.7%	14.3%	100.0%	.004
B	28	20	48	
	58.3%	41.7%	100.0%	

*Total*	76	28	104	

**Table 4:** Local infiltration (T) relative to recurrence-death.

Local infiltration	Recurrence-death			*P*-value
	No	Yes	*Total*	*Chi-square*
Submcosa-T1	10	1	11	
	90.9%	9.1%	100.0%	.012
Muscular propia-T2	38	7	45	
	84.4%	15.6%	100.0%	
Meso-T3	20	12	32	
	62.5%	37.5%	100.0%	
Serosa-T4	8	8	16	
	50.0%	50.0%	100.0%	

*Total*	76	28	104	

**Table 5:** Number of vessels relative to recurrence-death.

Number of objects	Recurrence-death			*P*-value
	No	Yes	*Total*	*Chi-square*
<37	47	20	67	
	70.1%	29.9%	100.0%	.500
≥37	29	8	37	
	78.4%	21.6%	100.0%	

*Total*	76	28	104	

**Table 6:** Percentage of vascular area relative to recurrence-death.

Vascular area %	Recurrence-death			*P*-value
	NO	YES	*Total*	*Chi-square*
<4	41	22	63	
	65.1%	34.9%	100.0%	.040
≥4	35	6	41	
	85.4%	14.6%	100.0%	

*Total*	76	28	104	
